# Solo Versus Tandem Cycling Performance: The Whole Is Less Than the Sum of the Parts

**DOI:** 10.1002/ejsc.70032

**Published:** 2025-08-22

**Authors:** Albert Smit, Julia Boschker, Stephan van der Zwaard, Ina Janssen, Thomas W. J. Janssen, Mathijs J. Hofmijster

**Affiliations:** ^1^ Department of Human Movement Sciences Faculty of Behavioural and Movement Sciences Vrije Universiteit Amsterdam the Netherlands; ^2^ Amsterdam Institute of Sport Science Amsterdam the Netherlands; ^3^ Koninklijke Nederlandsche Wielren Unie Arnhem the Netherlands; ^4^ Department of Cardiology Amsterdam University Medical Center University of Amsterdam Amsterdam the Netherlands; ^5^ Sport Science and Innovation Sportcentrum Papendal Arnhem the Netherlands

**Keywords:** cadence, interpersonal coordination, para‐cycling, power output, tandem cycling

## Abstract

Tandem cycling is a Paralympic discipline where two cyclists share one bicycle and requires synchronization and shared effort. In this study, we investigated how individual performance on a solo bicycle compares to tandem cycling. Twelve trained cyclists, that were unfamiliar with tandem cycling, completed a submaximal cycling test, a 30 s Wingate test, and a 10 min time trial under solo and tandem conditions. Performance metrics included gross efficiency, maximal power output, mean power output, heart rate, and cadence. The results showed that mean power output during the 10 min time trial was significantly lower on a tandem than on a solo bicycle (257 ± 66 W vs. 282 ± 80 W and *p* = 0.004), whereas cadence was higher (102.0 ± 7.7 rpm vs. 93.6 ± 4.7 rpm and *p* = 0.002). Gross efficiency, maximal power output, mean power output over 30 s, and heart rate over 10 min did not differ significantly between solo and tandem cycling. These findings suggest that although tandem cycling influences cadence and power, it does not reduce efficiency or short‐duration maximal performance. Given the differences in power output and cadence, coaches and athletes should carefully interpret solo cycling performance metrics when applied to tandem racing and training, especially in novice tandem cyclists. Future research should investigate the role of power output matching and tandem experience, and further explore the interaction between pilot and stoker to optimize performance outcomes.

## Highlights

- Cyclists that were unfamiliar with tandem cycling exhibit lower mean power output but higher cadence on a tandem during a 10 min time trial compared to solo cycling.
- Gross efficiency is not different between tandem and solo conditions despite differences in cadence and power output.
- Care should be taken when interpreting metrics obtained during solo cycling when analyzing tandem performance and prescribing training for tandem cycling, especially in novice tandem cyclists.

## 1 Introduction

Tandem cycling is a Paralympic discipline where a cyclist with a visual disability (the stoker; at the back) rides a tandem bicycle together with a cyclist without a visual disability (the pilot; at the front). The pilot is responsible for steering, braking, changing gears, and tactics, whereas the stoker needs to respond to the pilot's actions. Both athletes contribute to the total power output on the bicycle and ideally both perform at their maximum capacities, such that the combined power output is the sum of the maximum power output both cyclists can generate on an individual bicycle.

To date, and to the best of the authors' knowledge, only one study has compared solo and tandem cycling. Focusing on submaximal efforts, Seifert et al. (2003) assessed heart rate, lactate response, and perceived exertion at set velocities during both solo and tandem cycling on the road on a flat road course. Their participants were recreational road tandem cyclists, with male pilots and female stokers. They observed lower heart rate and lactate levels during tandem cycling for the stokers but observed no significant differences compared to solo cycling for the pilots. Being focused on submaximal intensities, this study provided limited insights into individual contributions at maximal intensities. Additionally, some international level tandem cyclists suggested that on a tandem, cyclists may produce lower maximal power compared to solo cycling (personal communication with A.S.).

The energy balance model for para‐cycling (Smit et al. 2024) describes performance as the interaction between power production, power dissipation, and gross efficiency. Power production is influenced by the athletes' physiological capacity, whereas power dissipation is affected by factors such as aerodynamics, rolling resistance, and drivetrain efficiency. In tandem cycling, additional complexities arise due to the need for synchronization between the pilot and stoker, potentially affecting cadence, power transfer, and overall efficiency. Gross efficiency, defined as the ratio of net external to internal power output, tends to rise with increasing power until it plateaus (de Koning et al. 2012). Factors, such as cadence and cycling position, influence gross efficiency (Marsh and Martin 1993; Price and Donne 1997; Sidossis et al. 1992), yet the impact of tandem cycling dynamics on gross efficiency remains unexamined. Tandem cycling's unique coordination demands, where cadence and phase of force production in the cyclic movement are connected between the two cyclists through mechanical coupling from the chain, could affect individual power output and efficiency. For example, power reductions have been reported in tandem cycling with Parkinson's patients due to stoker passiveness (Alberts et al. 2011), suggesting that power generated by one of the cyclists may potentially be dissipated by the other cyclist in some tandem settings, and thus be lost. However, in ideal situations where the two cyclists do not dissipate power from each other, the total power output will be the sum of the power output of the two cyclists on a tandem (Mohammadi‐Abdar et al. 2014). Alternatively, a physical connection between two people, such as the tandem's shared drivetrain, might lead to a better performance of either person, independent of the performance of the other person, due to haptic feedback (Ganesh et al. 2014). The physical connection on a tandem bicycle might, therefore, improve or hinder individual performance on a tandem, depending on the circumstances. As tandem cyclists usually train both alone and on a tandem with a fellow cyclist, it is essential to know whether there are differences in performance capacity between these two forms of cycling, when interpreting training data and in the selection of tandem partners.

The primary aim of this study was to examine how cycling with another cyclist on a tandem affects individual submaximal and maximal performance in tandem cycling. Specifically, we assessed gross efficiency, maximal and mean power output in a 30 s Wingate test, and mean power output, heart rate, and cadence during a 10 min time trial (TT) in both solo and tandem conditions. Secondary aims included examining the effect of rider position on potential differences in power output, cadence, and heart rate between solo and tandem cycling.

## 2 Materials and Methods

### 2.1 Participants

Twelve trained cyclists (3 females and 9 males; mean age 37.6 ± 15.1 years) volunteered for this study. Participants had an average of 15.3 ± 13.2 years of training experience, 3.3 ± 0.8 training sessions per week, and 7.1 ± 1.9 training hours per week, categorizing them as Tier 2 or “trained” athletes based on established guidelines (De Pauw et al. 2013; Decroix et al. 2016; McKay et al. 2022). None of the participants were visual impaired and the participants had no prior tandem cycling experience. Participants provided written informed consent after receiving information about the protocol and associated risks.

### 2.2 Experimental Design

All participants visited the laboratory on two occasions separated by a minimum of 48 h. Participants were instructed to refrain from vigorous physical activity for at least 24 h prior to each testing session. During the first lab visit, stature and body mass were measured using an electronic scale (seca robusta 813, seca, Germany) and stadiometer (HM‐250P Leicester Height Measure, Marsden, UK). Participants then performed several exercise tests on a tandem attached to computer‐controlled electromagnetically braked ergometer (Cyclus2, RBM electronic‐automation GmbH, Leipzig, Germany). The first test was a solo incremental graded submaximal exercise test, which served as a warm‐up for the subsequent tests and was used to calculate gross efficiency. Subsequently, all participants performed a 30 s Wingate anaerobic test (Wingate test) to determine maximal and mean power output over 30 s. Finally, they performed a 10 min TT to determine maximal mean power output, mean heart rate, and mean cadence. During the second visit, participants were randomly assigned a tandem partner and a role as either pilot or stoker. They performed the same tests as during the first visit, but this time with two persons on the tandem. Analogous to the first visit, the rear chain of the tandem was attached to the ergometer, albeit this time, the imposed load was doubled compared to the solo trial, such that the average load of each individual cyclist on the tandem was equal to the load imposed during the solo trials. This means that each cyclist in the tandem condition experienced an equivalent workload as they did in the solo condition, allowing direct comparison of physiological responses.

### 2.3 Data Collection

All tests were performed on a road tandem bicycle (Duratec Big Bang road, Duratec, s.r.o, Město Touškov, Czech Republic). The ergometer was powered by a direct current motor, with its axis replacing the rear wheel. A cassette was directly mounted to the motor axis and was driven by the rear chain of the tandem.

Individual power output was measured with power meter pedals (Garmin Rally RS200 Power meter, Garmin International Inc., Olathe, KS, USA). Their reliability was considered acceptable for determining power output (coefficient of variation from 2.9% to 5.05%) and cadence (coefficient of variation about 3.0%) (Dickinson and Wright 2020; Nimmerichter et al. 2017). Heart rate was measured with a chest strap (Garmin HRM‐Dual, Garmin International Inc., Olathe, KS, USA). Data of cadence, heart rate, and power output were sampled at 1 Hz on a cycle computer via the ANT + protocol (Edge 530, Garmin International Inc., Olathe, KS, USA). Expired air was analyzed breath‐by‐breath using open‐circuit spirometry (Quark CPET, Cosmed S.R.L., Rome, Italy) for the duration of the warming up. Before every test, the gas analyzer, volume transducer, and power meter pedals were calibrated according to manufacturer specifications.

For the solo tests, participants were seated at the position of the stoker at the rear of the tandem, and the secondary chain, which connects the crank of the pilot to the crank of the stoker, was removed to simulate a solo bicycle. The participants wore their own cycling shoes whereas saddle and handlebar height were adjusted to the preferences of the participant and kept constant during the tests. All experiments were conducted in an environmentally neutral laboratory (i.e., temperature 18.8 ± 1.2°C and relative humidity 48.3 ± 5.7%) at a similar time of the day for each participant.

### 2.4 Test Protocol

The submaximal test consisted of cycling at a freely chosen pedal rate, starting with 100 W for 3 min and increasing the power output every 3 min with 30 W for the solo test, and starting with 200 W with an average increase of 30 W per person for the tandem test, whereas measuring breath‐by‐breath pulmonary gas exchange to be able to determine gross efficiency. An incremental step duration of 3 min was chosen as pulmonary oxygen uptake increases mono‐exponentially before reaching a steady state within 2–3 min (Whipp and Wasserman 1972). Metabolic power was calculated from oxygen consumption and exhaled carbon dioxide as suggested by Garby and Astrup (1987). Gross efficiency was determined as the ratio between power output and metabolic power times 100%. Gross efficiency was established for stages where the respiratory exchange ratio (RER) ≤ 1.0 and the highest gross efficiency value was used in the analysis. The submaximal test served as the warming up for subsequent tests, which lasted at least 12 min. Incremental steps were continued until RER reached 1.05. If the step where the RER reached 1.05 ended before 12 min, the load remained at that level until the 12 min mark was reached. Otherwise, the test ended after the end of the step where the RER reached 1.05.

After 5 min of active recovery at 75 W for the solo test and a combined average of 75 W per person for the tandem test, participants performed a Wingate test, with the goal to achieve the highest mean power output as possible.

Using a torque factor of 0.7 and the crank length of 0.1725 m, the software of the ergometer calculated pedal force (in N) by multiplying body mass by 0.7 and dividing this by the crank length. This is analogous to Wingate tests performed on an ergometer where the flywheel is resisted by mechanical friction. A standardized verbal motivation was provided to encourage maximal effort in all Wingate sessions.

After another 10 min of active recovery at 75 W for the solo test and a combined average of 75 W per person for the tandem test, participants performed a 10 min TT, with the goal to cover as much virtual distance as possible. For the TT, the ergometer was set to “inclination” mode, which replicates real cycling dynamics, by simulating air resistance, rolling friction, gravity, and inertia. The mass of the rider(s) was given as input for each test. Simulated gears allowed the participants to control their own cadence during the TT by decreasing or increasing the simulated gear ratio by pressing buttons on the control panel of the ergometer. To minimize energy loss in transmission, the tandem's physical gear was set at 50 × 15 (Spicer et al. 2001). During the 10 min TT, visual feedback was provided and consisted of total power output (in W), cadence (in revolutions per min, rpm), and accumulated time and distance. For the tandem condition, this visual feedback was only visible for the pilot, where it described the combined effort of pilot and stoker. In this way, the feedback conditions during both solo and tandem time‐trials were closest to the real‐world situation. The slightly different feedback conditions in both time‐trials were not expected to influence the trial outcomes in a meaningful way, as Davies et al. (2016) showed in a meta‐analysis that the presence or absence of performance feedback has no significant effect on time‐trial performance. Furthermore, during the 10 min TT, participants were verbally informed about time completed at 2 min intervals by the test leader.

### 2.5 Statistics

Data are presented as mean ± standard deviation. Before any analysis, all data were checked for normality of distribution (Shapiro–Wilk) and equality of variances (Levene's). A 2 × 2 mixed‐design repeated measures ANOVA (RM ANOVA) was conducted, with bicycle type (solo vs. tandem) as the within‐subjects factor and tandem position (pilot vs. stoker) as the between‐subjects factor. Possible differences in maximal power output over 1 s, mean power output over 30 s, mean power output, cadence, heart rate during the 10 min TT, and gross efficiency between solo and tandem cycling were evaluated. Additionally, a bicycle × position interaction term was analyzed to assess whether the pilot or stoker role influenced differences in power output, heart rate, and cadence during the tandem tests. All statistical analyses were performed in JASP (Version 0.18.3.0), and statistical significance was accepted when *p* < 0.05. Effect sizes using omega squared (ω^2^) were calculated as this is a less biased measure for small sample sizes than with eta squared (Lakens 2013). Omega squared values range from 0 to 1.0 and can be interpreted as the amount of variance in the dependent variable that can be attributed to the independent variable.

## 3 Results

**FIGURE 1:**
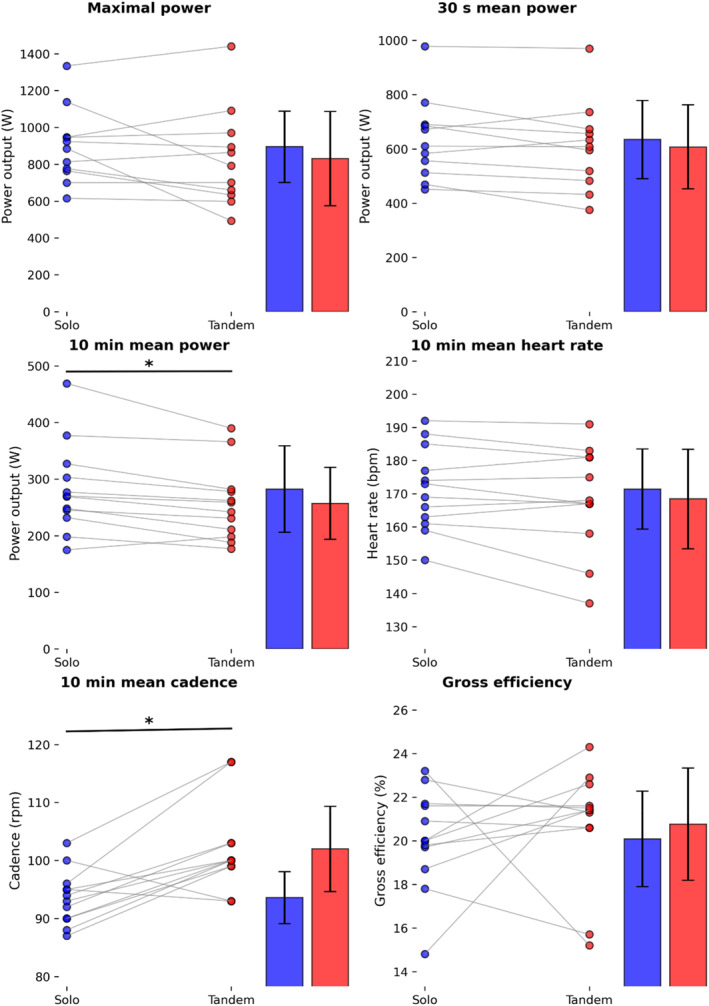
Solo and tandem cycling for maximal power over 1 s, mean power over 30 s, mean power over 10 min, mean heart rate over 10 min, mean cadence over 10 min, and gross efficiency * Indicated solo differs significantly from tandem (*p* < 0.01).

Data for maximal power, mean power, gross efficiency, mean heart rate, and mean cadence for solo and tandem cycling are shown in Figure 1. Due to incomplete data of the solo Wingate test of one participant, Wingate data of only 11 participants were analyzed. A Shapiro–Wilk test confirmed that the data were normally distributed, and Levene's test for equality of variances was not significant. Comparison between measured power output from the pedals and from the ergometer showed a mean difference of 3.7%, which can almost all be contributed to power losses in the chain drive (Smit et al. 2023).

Two significant effects were found when comparing solo to tandem cycling. Mean individual 10 min power output was significantly higher in solo cycling (282 ± 80 W vs. 257 ± 66 W, *p* = 0.006, and *ω* ^2^ = 0.028), whereas mean cadence was significantly lower in solo cycling (93.6 ± 4.7 rpm vs. 102.0 ± 7.7 rpm, *p* = 0.003, and *ω* ^2^ = 0.289). Although these results indicate statistically significant differences, the effect sizes provide important context for their practical relevance. The observed effect size for power output (*ω* ^2^ = 0.028) suggests a small effect, whereas the effect size for cadence (*ω* ^2^ = 0.289) is considerably larger, indicating a moderate‐to‐large effect. This suggests that changes in power and cadence due to tandem cycling are not only statistically significant but also practically relevant. No significant difference was found in gross efficiency between solo and tandem cycling (solo: 20.1 ± 2.3% and tandem: 20.8 ± 2.7%, *p* = 0.560, and *ω* ^2^ = 0.000). Given that ω^2^ was effectively zero, it is highly unlikely that a larger sample size would alter this conclusion. Similarly, the Wingate test data revealed no significant differences in maximal power output (solo: 895 ± 203 W and tandem: 830 ± 268 W, *p* = 0.253, and *ω* ^2^ = 0.006) and mean power output over 30 s (solo: 634 ± 151 W and tandem: 607 ± 162 W, *p* = 0.155, and *ω* ^2^ = 0.004) between solo and tandem cycling. In addition, mean heart rate did not differ significantly between the 10 min solo and tandem TT (solo: 171 ± 12.6 min^−1^ and tandem: 168 ± 15.6 min^−1^, *p* = 0.106, and *ω* ^2^ = 0.008).

Between‐subject effects, reflecting the influence of the pilot or stoker role, were not significant for any parameter (*p* > 0.05). Additionally, no significant bicycle × position interaction effects were observed for any parameter (*p* > 0.05 for all interaction terms). These results suggest that tandem position (pilot vs. stoker) did not affect any of the measured outcomes.

## 4 Discussion

To the best of our knowledge, this is the first study that investigated the effects of the presence of a fellow cyclist on individual maximal performance on a tandem bicycle. The main findings of the current study were that trained cyclists produced lower mean power output during a 10 min TT on a tandem bicycle with another cyclist compared to a solo bicycle, whereas no significant differences were found in power output between solo and tandem cycling during a 30 s Wingate test or in gross efficiency. Additionally, the cyclists adopted a significantly higher cadence in tandem cycling compared to solo cycling in the 10 min TT.

Our findings suggest that having a partner while cycling impacts power output and cadence in a continuous effort task which requires pacing, such as a 10 min TT, but not in a sprint task, such as a 30 s Wingate test. The submaximal test and 30 s Wingate test do not require any strategy and would therefore be easier to execute on a tandem, even without tandem experience. The power output–cadence relationship, described as an inverted U (Abbiss et al. 2009; Emanuele and Denoth 2012), might explain the decrease in power output with higher cadence in the 10 min TT. The power output–cadence relationship shows an increase in power output with an increase in cadence, up to a certain point, from where power output decreases with an increase in cadence. The observed mean cadence in the solo 10‐min TT of approximately 90 rpm aligns with optimal cadence reported in existing literature on highly trained cyclists (Foss and Hallen 2005; Lucia et al. 2001), whereas the mean cadence of approximately 102 rpm in the tandem 10min TT is higher than this optimal cadence, and probably therefore less optimal.

This increase in cadence may be better understood by considering the mechanical link between the pilot and stoker in tandem cycling, which necessitates synchronized pedaling. Similar coordination demands are observed in other activities where shared goals require precise timing, such as musical ensembles or cooperative sports (Sebanz et al. 2006). Research in interpersonal coordination, such as synchronized finger tapping, reveals that joint activities can increase movement speed, a phenomenon known as joint rushing (Okano et al. 2017; Thomson et al. 2018; Wolf and Knoblich 2022). The need for continuous perceptual adjustments between cyclists may drive a natural tendency toward a higher cadence, akin to synchronization effects seen in other coordinated tasks.

Building on this assumption, another possible explanation for the higher cadence and lower power output observed during the 10 min TT on the tandem could be the greater crank inertial load inherent in tandem cycling. The crank inertial load depends on the moment of inertia of the rotating parts, including the cranks, chains, and the ergometer axle. Since crank inertia increases as a quadratic function of the gear ratio (Fregly et al. 2000), it may lead cyclists to select higher cadences to reduce peak crank torque, thereby ensuring a smoother and more stable ride (Fregly et al. 1996; Hansen et al. 2002). As gear ratio was higher in the tandem 10 min TT than the solo 10 min TT, crank inertia might have influenced cadence.

Although the higher crank inertia supports consistent cadence, it poses a challenge for rapid acceleration such as during the Wingate test. Despite this, our study found nonsignificant differences in peak or mean power output between solo and tandem cycling in the Wingate test, with peak and mean power output in tandem cycling being lower than in solo cycling. However, some participants had higher power output in the Wingate on the tandem than during solo cycling. Challenges with rapid acceleration are seen in other sports with high inertia in team sprint performances, such as bobsleigh and sprint kayaking (Lopes and Alouche 2016; Ong et al. 2005). Studies in these sports highlight the importance of synchronized movements, especially at the start, and suggest that tandem cycling may benefit from similar insights into coordinated starts and the roles of team members (Campbell Ritchie and Selamat 2011; Lopes and Alouche 2016; Ong et al. 2005; Romagnoli et al. 2024). It could be that some participants were not synchronized enough on the tandem to produce the same power output as solo, whereas others were able to do that.

Turning to gross efficiency, no significant differences were observed between solo and tandem cycling, suggesting that the efficiency of energy use remains consistent across both conditions. However, although gross efficiency can be influenced by various factors, such as cadence, riding position, and environmental conditions (Chavarren and Calbet 1999; Heil et al. 1995; Hettinga et al. 2007; Marsh and Martin 1993; Nimmerichter et al. 2015; Price and Donne 1997; Sidossis et al. 1992), tandem cycling itself did not elicit a mean difference in this metric. Notably, large intraindividual variations in gross efficiency were linked to differences in power output between the solo and tandem conditions, warranting cautious interpretation, particularly for teams with significant interindividual power discrepancies.

Our findings are in contrast with those from Seifert et al. (2003), who reported lower physiological stress in stokers during tandem cycling compared to solo conditions, whereas the absence of an (interaction) effect between bicycle type and tandem position in our study did not indicate such an effect. In their study, pilots experienced similar heart rate, blood lactate, and perceived exertion in solo and tandem trials at the same velocity, whereas the stokers had a lower heart rate, blood lactate, and perceived exertion on the tandem at all velocities compared to solo cycling. The differences in our findings may be due to the trained status of our participants compared to the recreational cyclists studied by Seifert et al. (2003). Trained cyclists may have a greater ability to sustain effort under different conditions, which could explain the absence of significant differences in physiological stress between pilots and stokers in our study.

However, regarding relative intensity in pilot and stoker on the tandem, we found similar results as Seifert et al. (2003). They found no significant differences in perceived exertion on the tandem between pilot and stoker, as we found no significant differences in heart rate between solo and tandem cycling for pilot and stoker on the tandem, and as heart rate is highly correlated with perceived exertion (Gillach et al. 1989), pilot and stoker were probably experiencing similar relative intensity as on the solo test. This implies that social loafing, also known as the Ringelmann effect (Ingham et al. 1974), appeared not to be present in our study or in the study by Seifert et al. (2003). A lower heart rate or rating of perceived exertion would be expected if the effort of pilot or stoker would have been lower than their partner. It can be hypothesized that particularly stokers are susceptible to such a phenomenon, as social loafing is usually more present when the performance goes unnoticed (Harkins 1987), and on a tandem the stoker can not be seen by the pilot. However, increased awareness of each other's effort, such as through shared performance feedback, could potentially foster social facilitation (Harkins 1987; Triplett 1898), the opposite of social loafing, where performing alongside another person increases the effort. Social facilitation trough shared performance feedback could enhance tandem performance (Harkins 1987).

### 4.1 Limitations

This study provides valuable insights into tandem cycling performance; however, some limitations should be considered when interpreting the findings. None of the participants had prior tandem cycling experience, and no instructions were given on how to cycle on a tandem, which may have influenced their ability to synchronize effectively. More experienced tandem cyclists may exhibit improved synchronization, potentially reducing the power loss observed in our study. Future studies should include experienced tandem cyclists to better understand to what extent familiarity and practice impact performance. Furthermore, athletes were paired randomly without considering their power output compatibility, which might have affected the synchronization and overall performance. Future studies could systematically pair cyclists based on similar or mismatched cadence preferences to assess the impact of optimal partner matching on performance. Additionally, the testing sequence always began with solo cycling, potentially introducing order effects that could influence performance comparisons. Counterbalancing the order of solo and tandem tests in future studies is recommended to minimize this bias. However, despite the possible order effect, power output was still significantly lower in the tandem TT than the solo TT. Finally, the cadence was not standardized during the submaximal test, which might have influenced the determination and comparison of gross efficiency.

## 5 Practical Applications

Given the performance differences in the 10 min time trial—but not in the Wingate test—coaches should be cautious when comparing power output in solo and tandem cycling, particularly for novice tandem cyclists. Solo efforts may yield higher power output than tandem efforts over the same duration, making solo‐based performance goals unrealistic for tandem events. Coaches should set performance targets specific to tandem cycling, accounting for these differences. Moreover, scouts that test novice tandem cyclists in solo tests should be aware of reduced power output values during tandem cycling with another cyclist.

## 6 Conclusion

This study demonstrates that trained cyclists produce significantly lower power output and higher cadence during a 10 min time trial on a tandem bicycle compared to solo cycling when they are unfamiliar with tandem cycling. However, gross efficiency and sprint performance, as measured by the 30 s Wingate test, remained similar between the two conditions. These findings suggest that tandem cycling directly affects cadence and endurance performance without compromising efficiency or short‐duration maximal efforts. Therefore, coaches and athletes should be cautious when applying solo cycling performance metrics to tandem scenarios in novice tandem cyclists, given the unique coordination demands of tandem cycling. Future research should examine the impact of tandem experience and the interaction between pilot and stoker on power output and pacing strategy to further optimize tandem performance.

## Ethics Statement

Ethical approval was granted by the Faculty of Behavioral and Movement Sciences, Vrije Universiteit Amsterdam (VCWE‐2024‐057).

## Conflicts of Interest

The authors declare no conflicts of interest.
